# Linguistic and psychometric validation of the MSSS-88 questionnaire for patients with multiple sclerosis and spasticity in Germany

**DOI:** 10.1186/s12955-014-0119-y

**Published:** 2014-08-01

**Authors:** Thomas Henze, Sylvia von Mackensen, Gerald Lehrieder, Uwe K Zettl, Carmen Pfiffner, Peter Flachenecker

**Affiliations:** Passauer Wolf Rehabilitation Center Nittenau, Nittenau, Germany; Institute of Medical Psychology, University Medical Centre Hamburg-Eppendorf, 20246 Hamburg, Germany; Neurological Department of Dr. Becker Klinikgesellschaft mbH & Co. KG, Dr. Becker Kiliani-Klinik, Bad Windsheim, Germany; University of Rostock, Center of Neurology, Rostock, Germany; IMS Health, Munich, Germany; Neurological Rehabilitation Center Quellenhof, Bad Wildbad, Germany

**Keywords:** MSSS-88, Linguistic validation, Psychometric evaluation, Multiple sclerosis, Spasticity, Patient-reported outcome

## Abstract

**Background:**

Multiple sclerosis (MS) is an inflammatory disease where many of the patients suffer from spasticity impacting their quality-of-life. The purpose of this paper was to linguistically validate and psychometrically test the Multiple Sclerosis Spasticity Scale (MSSS-88) in German speaking MS patients.

**Methods:**

The study had two stages: 1) forward/backward translations of the original MSSS-88 scale into German, discussions with MS-experts and cognitive debriefings with MS patients; 2) psychometric evaluation of the German version. Data collection took part in an observational multi-centre study in Germany (MOVE2).

**Results:**

The German translation of the MSSS-88 scale was discussed with three MS-experts; followed by two cognitive debriefing sessions with 12 MS patients. For psychometric evaluation the MSSS-88 was filled in by 87 MS patients with a mean age of 50.2 ± 10.4 years; 26.4% of them had severe spasticity. Data quality was acceptable. Missing data for items of the MSSS-88 were low (range 0–5.75%). Psychometric testing of the MSSS-88 revealed excellent values for reliability and validity. Significant differences between groups regarding severity, grading, type and self-ratings of MS-spasticity and sleep disturbances were found. Sensitivity to change could be demonstrated for the MSSS-88 in the group of MS patients treated with cannabinoid oromucosal spray vs. non-treated patients. In the treated group significant changes with a moderate effect size were found for ‘muscle spasms’, ‘emotional health’ and ‘pain/discomfort’. No significant changes could be detected in the non-treated group.

**Conclusion:**

Preliminary evidence from this small study supports reliability, validity, and responsiveness of the German version of the MSSS-88 for measuring the impact of spasticity in MS.

## Background

Multiple sclerosis (MS) is an inflammatory disease of the brain and spinal cord resulting in neurological dysfunction [1]. MS-symptoms can occur in episodic attacks (relapsing form), or gradually accumulate over time (progressive form), or in a combination of both [2]. MS patients typically suffer from the following neurological symptoms or signs: changes in sensation (e.g. loss of sensitivity, tingling, pricking or numbness), muscle weakness, muscle spasms, difficulties in mobility and coordination, visual, speech and swallowing problems, bladder and bowel disturbances, or pain; in addition, fatigue, cognitive impairments and affective symptoms may also often occur [3-7]. Up to 90% of MS patients suffer from spasticity [8], which can be defined as “a motor disorder characterized by a velocity-dependent increase in tonic stretch reflexes (muscle tone) with exaggerated tendon jerks resulting from hyperexcitability of the stretch reflex as one component of upper motor neuron syndrome” [9]. MS patients refer to spasticity-related symptoms using a range of terms including muscle rigidity, sudden jerks or contractions in limbs or trunk often associated with pain [10]. The severity of spasticity tends to increase with the progression of the disease [11]. Spasticity can significantly affect motor performance, activities of daily living, and well-being [12-14]. It can be measured via electrophysiological, biomechanical and clinical evaluation [15]. For clinical evaluation of the severity of spasticity in MS the (Modified) Ashworth scale remains the most widely used by health specialists [16,17], together with the categorical severity scale (mild/moderate/severe) [18]. Recently the patient-reported numeric rating scale (NRS) has been used for the subjective assessment of the severity of spasticity from the patient’s perspective [19,20]. Progression of disability and severity of impairment in MS patients can be measured with the Expanded Disability Status Scale (EDSS) [21,22], which is recommended in the German guideline for use in clinical-neurological examination of MS [23].

In the last decades, there has been a growing interest in including patient-reported outcomes (PROs) in medical research [24]. Several disease-specific PRO instruments assessing the impact of the disease on MS patients have been developed in recent years. An overview of validated instruments is given in a review paper by Opara et al. [25]. Recently, the **Multiple Sclerosis Spasticity Scale (MSSS-88)** has been developed with the aim of constructing a self-assessment instrument to measure patients’ perception and experiences of the impact of spasticity due to MS. It was developed to have an additional instrument to existing neurophysiological, clinical and biomedical techniques lacking to measure the broader consequences of spasticity for the patient. The MSSS-88 scale consists of 88 items pertaining to 8 domains: ‘muscle stiffness’, ‘pain/discomfort’, ‘muscle spasms’, ‘activities of daily living’ (ADL), ‘walking’, ‘body movements’, ‘emotional health’ and ‘social functioning’. Each item is rated on a 4-point scale with answers from 1 (‘not at all bothered’) to 4 (‘extremely bothered’) [26]. Subscale values are transformed from 0-100 with high scores indicating high impairment allowing comparison across subscales. The MSSS-88 provided solid psychometric properties in the study sample, but was only available in English up to now. To use the MSSS-88 in German patients, linguistic validation is necessary. Linguistic validation and cross-cultural adaptation of PRO instruments should follow established procedures [27-29], which are based on the *translation* (including forward translation, reconciliation, back translation and cognitive debriefing) of the original language into a target language, followed by the *psychometric testing* of the instrument in the new population.

The aim of this project was to linguistically validate and cross-culturally adapt the original MSSS-88 questionnaire from English into German for the use in German speaking MS patients with spasticity and testing its psychometric characteristics.

## Patients and methods

### Design

The study had two stages including 1) the development of the MSSS-88 German version, and 2) data collection in German MS patients and psychometric evaluation.

#### Stage 1: Development of the MSSS-88 German version

The MSSS-88 was translated from English into German by two independent translators experienced in medical translation. Differences in the two resulting versions were discussed with a MS-expert and reconciled by two members of the study group (SvM, CP). After back-translation of the consensus version by one independent translator, the original MSSS-88 English scale and the English back-translation were compared. Three MS-experts were asked to discuss phrasing and content of all items. Suggestions were implemented and the modified German version was discussed with MS patients in two cognitive debriefing (CD) sessions (four and eight patients, informed consent prior to CD). MS experts and MS patients discussed each item and gave recommendations for linguistic improvement or rewording of unclear items. Items which caused problems (e.g. ambiguity, wording) were discussed with two of the developers of the original MSSS-88 scale (J.C. Hobart, A. Riazi) to ensure the original item concepts were maintained and to clarify unclear aspects.

#### Stage 2: Psychometric evaluation

For the psychometric evaluation (PE) of the MSSS-88 we had the chance to include und test the new German translation of the MSSS-88 in the frame of the German-wide multicentre, prospective, observational, non-interventional MOVE2 study (Mobility Improvement in MS patients with spasticity) [30] as an ancillary study in a subset of the enrolled MS patients. The study was approved by the Landesärztekammer (State Medical Association) Baden-Württemberg and the ethics committee of the medical faculty of the University of Rostock in Germany. The aim of the MOVE2 study was to evaluate the clinical outcomes and safety of a cannabinoid oromucosal spray in the real-life setting after its launch in Germany.

### Study centers and patients

A total number of 10 out of the 42 participating study centers from the MOVE2 study (office-based neurologists, MS outpatient clinics, rehabilitation centres, neurological hospitals) were asked to recruit at least 10 eligible MS patients with spasticity resulting in a planned total sample of 100 MS patients (1/3 mild, 1/3 moderate, 1/3 severe). Additional patients with mild spasticity were recruited in order to include the full range of patients beyond the inclusion criteria of the MOVE2 study. A formal sample size calculation was not carried out prior to the study. The sample size was informed by sample sizes used in published validation studies as well as based on the availability and eligibility of patients in the study centers of the MOVE2 study who were willing to participate. Patients were eligible when they had fulfilled the following inclusion criteria: age ≥18 years, MS-diagnosis with mild, moderate or severe spasticity (severity was rated by the physician, defined as whenever spasticity causes limitations to ADL, activities in a social environment or where there is a danger of spasticity-related complications), patient’s informed consent. No restriction was done related to spasticity treatment. Exclusion criteria were: spasticity due to other causes than MS, patients with relevant cognitive impairment due to physicians’ judgment or deficits of the German language. Patients were consecutively recruited by the participating physicians due to the point of time of their regular visit and when they met the inclusion criteria. Some of the participating patients started treatment with an oromucosal cannabinoid spray as add-on therapy to their already existing antispastic drug therapy if their spasticity was moderate to severe and they had further existing spasticity symptoms.

### Data collection

Patients filled in the MSSS-88 scale together with other PRO measures; clinical data were collected from physicians. Patients filled in the questionnaires twice (at inclusion visit and as a postal data collection after one month (FU1)), including demographic and clinical characteristics as well as validated instruments assessing HRQoL with the generic EQ-5D-3L (five domains: ‘mobility’, ‘self care’, ‘usual activities’, ‘pain/discomfort’, ‘anxiety/depression’) [31,32] and the MS-specific MSQoL-54 (consisting of 54 items pertaining to 12 subscales and two composite scores: ‘physical health’, ‘mental health’) [33,34]. Patients rated the degree of spasticity on the 11 point (0 to 10) NRS [19] and filled in the German “Würzburger Fatigue Inventory for MS” (WEIMuS), consisting of 17 items pertaining to 2 domains (‘physical fatigue’, ‘cognitive fatigue’) [35]. Physicians filled in the chart documentation form (CDF) at inclusion including demographics, clinical MS-characteristics, concomitant diseases, treatment with MS drugs and antispastic drugs, mobility, treatment setting (outpatient, inpatient rehabilitation, inpatient hospital) and rated the severity of spasticity (mild, moderate, severe). They also rated the global degree of spasticity (not specified for specific muscles) using the Modified Ashworth 5 level scale [17], the disability in EDSS [21,36], and ADL in Barthel Index [37,38].

### Statistical analysis

Data are presented as means, standard deviations (SD) and ranges or in percentages. Spearman correlation coefficients were conducted; and group differences were calculated by means of the Student’s T-test, Wilcoxon-signed-rank-test or ANOVA according to distribution of parameter values and number of groups compared. Patients without treatment with cannabinoid oromucosal spray were classified as “standard group” (no changes of patient’s status due to medication were expected between baseline and FU1). Sensitivity to change analysis was conducted in the subgroup of patients receiving cannabinoid oromucosal spray (“treatment group”, an improvement of spasticity situation was expected since they had an add-on anti-spastic therapy). The PE of the German version of the MSSS-88 scale included testing on data quality, scaling assumption, floor and ceiling effects, MSSS-88 subscale inter-correlation, reliability, construct validity and responsiveness. We used only classical test theoretical methods to replicate the psychometric characteristics of the MSSS-88 scale instead of applying as well probabilistic methods such as the Rasch model since a) both methods produce similar results, b) Rasch models need large sample sizes (minimum 200), which we did not have in our study c) Rasch models are complicated and not widely used as mentioned as well by the authors of the MSSS-88 [26].

#### Reliability

*Internal consistency* was estimated with Cronbach’s α coefficient and *test-retest reliability* was analyzed with Intra-Class Correlation Coefficient (ICC) between subscale values at baseline and at FU1 (two-way random–effects model). The upper limit of two months for the time period between the two visits was determined.

#### Construct validity

*Convergent validity* was calculated via correlations between MSSS-88 subscales with corresponding subscales of other PRO-instruments (MSQoL-54, EQ-5D-3L, WEIMuS) measuring similar constructs; ‘moderate’ (r = 0.41–0.60) to 'high' correlation coefficients (r = 0.61-0.80) (p < 0.05) were expected [39]. *Discriminant validity* was investigated by correlations between MSSS-88 subscale values with subscales of WEIMuS; low correlation coefficients were expected. *Criterion validity* was explored by correlations of MSSS-88 subscale values with well-respected outside measures (Barthel Index, Modified Ashworth Scale, NRS spasticity and EDSS) [40]. The goal of *known groups validity analysis* was to determine whether MSSS-88 is able to detect expected differences across patient subgroups for the following clinical characteristics: type of spasticity, physicians’ grading of spasticity (Modified Ashworth Scale: low [0-2] vs. high [3,4]; severity of MS-spasticity (mild, moderate, severe)), and patients’ grading of NRS spasticity (low [<5] vs. high [≥5]) and sleep disturbances (low [<3] vs. high [≥3]).

#### Responsiveness

Responsiveness was explored by comparing the change scores and effect sizes in the MSSS-88 subscales between baseline and FU1 (range 2 weeks to 2 months) in two patient groups: ‘treated’ patients and ‘standard group’. It was expected that ‘treated’ patients would report significant improvements across the MSSS-88 subscales (especially for ‘muscle spasm’ and ‘pain/discomfort’) and the NRS, while no significant changes were expected in the “standard group”. Effect size (Cohen’s *d)* was measured by the standardized mean difference calculated as mean difference between baseline and FU1 divided by the SD of the difference [41]. Effect sizes of *d* = 0.2 are indicating small effects, *d* = 0.5 medium and *d* = 0.8 large effect sizes [41]. An additional analysis was conducted with patients who were classified as “responders”, defined as improvement ≥20% on the patient-rated NRS scale between baseline and FU1 with accordance to the definition applied by Novotna [20]. It was expected that “responders” would report significantly better values in the MSSS-88 subscales. All statistical analyses were conducted on 0.05 significance level using SAS version 9.2.

## Results

### Stage 1 (linguistic validation)

The comparison of the back-translation and the original MSSS-88 questionnaire identified 19 items requiring refinement, which were discussed with key MS opinion leaders. From the expert discussion, 6 items (no. 13, 17, 28, 41, 55, 61) were considered as needing to be further discussed with the patients concerning their formulation. Two CD-sessions were conducted with 12 MS patients (9 women; mean age 55.8 ± 12.1 years) with all types of severity and most of them with restricted mobility. Completion time of the German MSSS-88 was in average 20 minutes. Unclear aspects were clarified with the developers of the MSSS-88, e.g. “difficulties moving freely”, which was not understood consistently by patients (possible interpretation by patients were ‘without walking aid’, ‘unrestricted/unhindered’ or related to ‘the distance they can walk’). The final instrument was well accepted and understood by patients.

### Stage 2 (psychometric evaluation)

Eight study centers enrolled patients; 2 centers did not participate due to organizational reasons. In total, 90 patients gave informed consent and participated in the period 08/2011 to 03/2012. Valid questionnaires were available from 87 patients at inclusion, 66 patients sent their questionnaire back at FU1. At inclusion, 36 patients (moderate: 20, severe: 16) from the MOVE2 study participated in the MSSS-88 PE and 51 patients (mild: 37, moderate: 7, severe: 7) were recruited additionally. Socio-demographic and clinical data are summarized in Table 1. The major part of patients was female, had a mild form of spasticity and was outpatients. At inclusion 36 patients (41.4%) started treatment with cannabinoid oromucosal spray.

**Table 1:** **Socio-demographic and clinical characteristics of MS patients at inclusion**

**Characteristics**	**Total number of patients**	**Patients treated with cannabinoid oromucosal spray**	**Patients** ***not*** **treated with cannabinoid oromucosal spray**
	**n = 87**	**n = 36**	**n = 51**
**Socio-demographic data**			
Age M ± SD (range)	50.2 ± 10.4 (27-73)	51.1 ± 11.3 (27-73)	49.5 ± 9.8 (28-71)
Gender (female) n (%)	54 (62.1)	20 (55.6)	34 (66.7)
Employed* n (%)	26 (30.2)*	10 (27.8)	16 (32.0)*
Living situation*			
Alone n (%)	16 (18.6)	7 (19.4)	9 (18.0)
with family/ partner n (%)	67 (77.9)	27 (75.0)	40 (80.0)
Nursing home n (%)	1 (1.2)	0 (0.0)	1 (2.0)
Other n (%)	2 (2.3)	2 (5.6)	0 (0.0)
**Clinical data**			
**Parameters physician report**			
Duration of multiple sclerosis in years since onset M ± SD (range)	13.6 ± 8.4* (0.2-36)	14.3 ± 8.9 (3-36)	13.1 ± 8.1* (0.2-34)
Type of multiple sclerosis			
Primary progressive n (%)	16 (18.4)	7 (19.4)	9 (17.7)
Secondary progressive n (%)	44 (50.6)	15 (41.7)	29 (56.9)
Relapsing remitting n (%)	27 (31.0)	14 (38.9)	13 (25.5)
Mean number of MS relapses in the last 12 months M ± SD (range)	1.4 ± 0.7 (1-4)	1.9 ± 1.1 (2-4)	1.1 ± 0.3 (1-2)
Duration of spasticity in years since onset M ± SD (range)	8.0 ± 6.6** (0.2-27)	8.2 ± 5.6 (1.0-21)	7.9 ± 7.4** (0.2-27)
EDSS M ± SD (range)	5.7 ± 1.6 (1.5-8.5)	5.8 ± 1.7 (2.0-8.5)	5.6 ± 1.5 (1.5-8.5)
Type of spasticity (physician report)			
Persistent n (%)	46 (52.9)	17 (47.2)	29 (56.9)
Paroxysmal n (%)	31 (35.6)	12 (33.3)	19 (37.2)
Both n (%)	10 (11.5)	7 (19.5)	3 (5.9)
Severity of spasticity (physician report)			
mild n (%)	37 (42.5)	0 (0.0)	37 (72.5)
moderate n (%)	27 (31.0)	21 (58.3)	6 (11.8)
severe n (%)	23 (26.5)	15 (41.7)	8 (15.7)
Modified Ashworth Score M ± SD (range)	2.3 ± 1.1*** (0-4)	3.0 ± 0.8* (1-4)	1.7 ± 1.0* (0-4)
Barthel index M ± SD (range)	71.2 ± 27.5 (0-100)****	71.0 ± 29.5 (0-100)	71.7 ± 21.6 (25-95)****
Concomitant symptoms			
Fatigue n (%)	67 (77.0)	30 (83.3)	37 (72.6)
Cognitive disorder n (%)	21 (24.1)	13 (36.1)	8 (15.7)
Depressive mood n (%)	18 (20.7)	9 (25.0)	9 (17.7)
Mobility			
unaided n (%)	29 (33.3)	9 (25.0)	20 (39.2)
crutches n (%)	16 (18.4)	8 (22.2)	8 (15.7)
walking frame n (%)	14 (16.1)	3 (8.3)	11 (21.6)
wheelchair n (%)	28 (32.2)	16 (44.5)	12 (23.5)
Treatment with immunomodulators in the past 12 months n (%)	18 (20.7)	20 (55.6)	43 (84.3)
Drug treatment of MS-spasticity at inclusion n (%)	57 (65.5)	27 (75.0)	30 (58.8)
**Parameters patient report**			
Level of spasticity (NRS) M ± SD (range)	5.1 ± 2.4 (0-10)	6.5 ± 1.7 (4-10)	4.0 ± 2.3* (0-9)
Level of sleep disturbances (NRS) M ± SD (range)	3.2 ± 2.9* (0-10)	4.3 ± 3.4 (0-10)	2.4 ± 2.2 (0-8)

*1 patient with missing data; **5 patients with missing data; ***2 patients with missing data; ****38 patients with missing data.

### Score distributions of the MSSS-88 at inclusion

Missing data for items of the MSSS-88 were low (range 0–5.75%); all 88 items were answered by 52 patients. Subscales could be calculated for all patients indicating that data quality was acceptable. Item means ranged from 1.43 to 3.28 and SDs from 0.68 to 1.17 (Table 2). Corrected item-total correlations exceeded 0.40 with the vast majority exceeding 0.60. Patients reported high baseline impairments in the subscale ‘walking’ (65.7 ± 25.6), ‘muscle stiffness’ (59.3 ± 25.6) and ‘body movements’ (58.8 ± 28.2). Low floor effects were found for ‘walking’ (8.6%) and ’body movements (7.1%), indicating that some patients reported high impairments in the respective scales, while low ceiling effects could be demonstrated for ‘ADL’ (7.1%) and ‘social functioning’ (7.1%), indicating that some patients had no impairments in all aspects of the respective scales (Table 2). Inter-correlations among MSSS-88 subscales showed high values for ‘body movement’ and ‘walking’ (r = 0.80), for ‘social functioning’ and ‘emotional health’ (r = 0.78) as well as for ‘muscle stiffness’ and ‘muscle spasm’ (r = 0.75).

**Table 2:** **MSSS-88 score distributions and reliability [at inclusion (n = 87), and FU1 for test-retest (n = 66)]**

**MSSS-88 subscale**	**No of items**	**Items mean (range)**	**Items SD (range)**	**Item total correlation**	**Scale Mean ± SD (range)**	Floor effect* %	Ceiling effect* %	**Cronbach’s alpha**	Cronbach’s alpha original MSSS-88**	Test-retest ICC*** (n = 66)
Muscle Stiffness	12	2.09 - 3.07	0.82 - 1.04	0.67 - 0.83	59.3 ± 25.6 (5.6 – 100.0)	2.3	0.0	0.95	0.95	0.74
Pain and Discomfort	9	1.90 - 2.66	0.91 - 1.10	0.55 - 0.81	43.3 ± 26.7 (0.0 – 100.0)	1.2	2.4	0.92	0.95	0.67
Muscle Spasms	14	1.43 - 2.61	0.68 - 1.15	0.42 - 0.75	39.6 ± 23.9 (0.0 – 92.3)	0.0	2.3	0.92	0.93	0.47
Activities of Daily Living	11	2.16 - 2.74	1.03 - 1.17	0.81 – 0.90	47.1 ± 31.8 (0.0 – 100.0)	6.0	7.1	0.97	0.95	0.87
Walking	10	2.82 - 3.21	0.83 - 1.02	0.63 - 0.87	65.7 ± 25.6 (13.3 – 100.0)	8.6	0.0	0.95	0.96	0.67
Body Movements	11	2.46 - 3.28	0.83 - 1.11	0.78 - 0.87	58.8 ± 28.2 (6.1 – 100.0)	7.1	0.0	0.96	0.96	0.72
Emotional Health	13	1.54 - 2.36	0.77 - 1.04	0.69 - 0.84	34.1 ± 25.6 (0.0 – 94.9)	0.0	1.2	0.95	0.96	0.61
Social Functioning	8	1.74 - 2.63	0.87 - 1.09	0.66 - 0.84	36.6 ± 28.1 (0.0 – 100.0)	1.2	7.1	0.94	0.95	0.62

*Ceiling and floor effects are the percent of people scoring the best (ceiling, score = 0) or worst (floor, score = 100) possible*.*

**Hobart et al. [26].

***Intra-Class Correlation Coefficient (ICC).

### Reliability

In terms of **internal consistency** Cronbach’s alpha coefficients ranged from 0.92 to 0.97, and exceeded the recommended minimum value of 0.70 [42] (Table 2). Cronbach’s alpha coefficients were similar to those reported for the original MSSS-88 [26]. For the **test-retest reliability** ICC coefficients are shown in Table 2 for each subscale of 66 eligible patients who filled in the questionnaire twice. Three of eight subscales had values over 0.7, and four subscales had values between 0.6 and 0.7.

### Construct validity

#### Convergent and discriminant validity

Most correlations between MSSS-88 subscales with corresponding subscales had a magnitude and pattern as expected (Table 3). Spearman correlations indicated convergent validity with values ranging from r = 0.40 to r = 0.73, with high correlations of MSSS-88 subscales with corresponding subscales of the disease-specific MSQoL-54. Discriminant validity could be proven by low correlations of the MSSS-88 scales with scales of the WEIMuS Inventory.

**Table 3:** **Convergent and discriminant construct validity [at inclusion (n = 87)]**

**MSSS-88 Subscale**	**Convergent Validity**			**Discriminant Validity**		
	**Instrument**	**Subscale**	**Spearman correlation coefficient**	**Instrument**	**Subscale**	**Spearman correlation coefficient**
**Muscle Stiffness**	EQ-5D	Mobility	0.40	WEIMuS	Cognitive fatigue	0.28
	MSQoL-54	Physical health	0.56			
**Pain and Discomfort**	EQ-5D	Pain/discomfort	0.58	WEIMuS	Cognitive fatigue	0.44
	MSQoL-54	Pain	0.71			
**Muscle Spasms**	EQ-5D	Mobility	0.49	WEIMuS	Cognitive fatigue	0.26
	MSQoL-54	Physical health	0.52			
**Activities of Daily Living**	EQ-5D	Usual activity	0.68	WEIMuS	Cognitive fatigue	0.25
	MSQoL-54	Physical health	0.71			
**Walking**	EQ-5D	Mobility	0.44	WEIMuS	Physical fatigue	0.34
	MSQoL-54	Physical health	0.68			
**Body Movements**	EQ-5D	Mobility	0.59	WEIMuS	Cognitive fatigue	0.26
	MSQoL-54	Physical health	0.67			
**Emotional Health**	EQ-5D	Anxiety/depression	0.65			
	MSQoL-54	Emotional well-being	0.69			
		Health distress	0.73			
**Social Functioning**	WEIMuS	Cognitive Fatigue	0.58			
	MSQoL-54	Social function	0.61			

Spearman correlation between MSSS-88 subscales and corresponding subscales of other patient-reported instruments.

#### Criterion validity

Acceptable correlations were found for the MSSS-88 subscales ‘muscle stiffness’ and ‘muscle spasm’ and the global rating of the Modified Ashworth Scale, the NRS and the EDSS, as well as for the subscale ‘ADL’ and the Barthel Index, the Modified Ashworth Scale, the NRS and the EDSS and for the subscale ‘body movement’ and the EDSS (Table 4).

**Table 4:** **MSSS-88 criterion validity [at inclusion (n = 87)]**

**MSSS-88 subscale**	**Modified Ashworth scale**	**Barthel index**	**NRS spasticity**	**EDSS**
Muscle Stiffness	0.52	0.45	0.53	0.45
Pain and Discomfort	0.26	0.14	0.52	0.27
Muscle Spasms	0.50	0.47	0.63	0.50
Activities of Daily Living	0.58	0.82	0.56	0.64
Walking	0.35	0.56	0.38	0.44
Body Movements	0.53	0.66	0.51	0.55
Emotional Health	0.24	0.09	0.36	0.12
Social Functioning	0.29	0.30	0.48	0.37

Spearman Correlations with Modified Ashworth Scale, Barthel Index, NRS spasticity and EDSS measures.

#### Known groups’ validity

**Figure 1:**
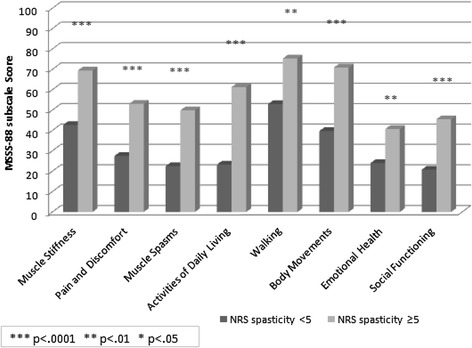
**Mean MSSS-88 subscale score for people with high (NRS ≥ 5, n = 54) and low (NRS < 5, n = 32) self-reported NRS spasticity.** NRS: numerical rating scale (0 – 10).

Significant differences were found between patients with *low vs. high self-reported NRS spasticity* (Figure 1); patients with high NRS spasticity reported higher impairments in all MSSS-88 subscales. The same pattern was shown for the self-reported level of sleeping disturbances (data not shown).

**Figure 2:**
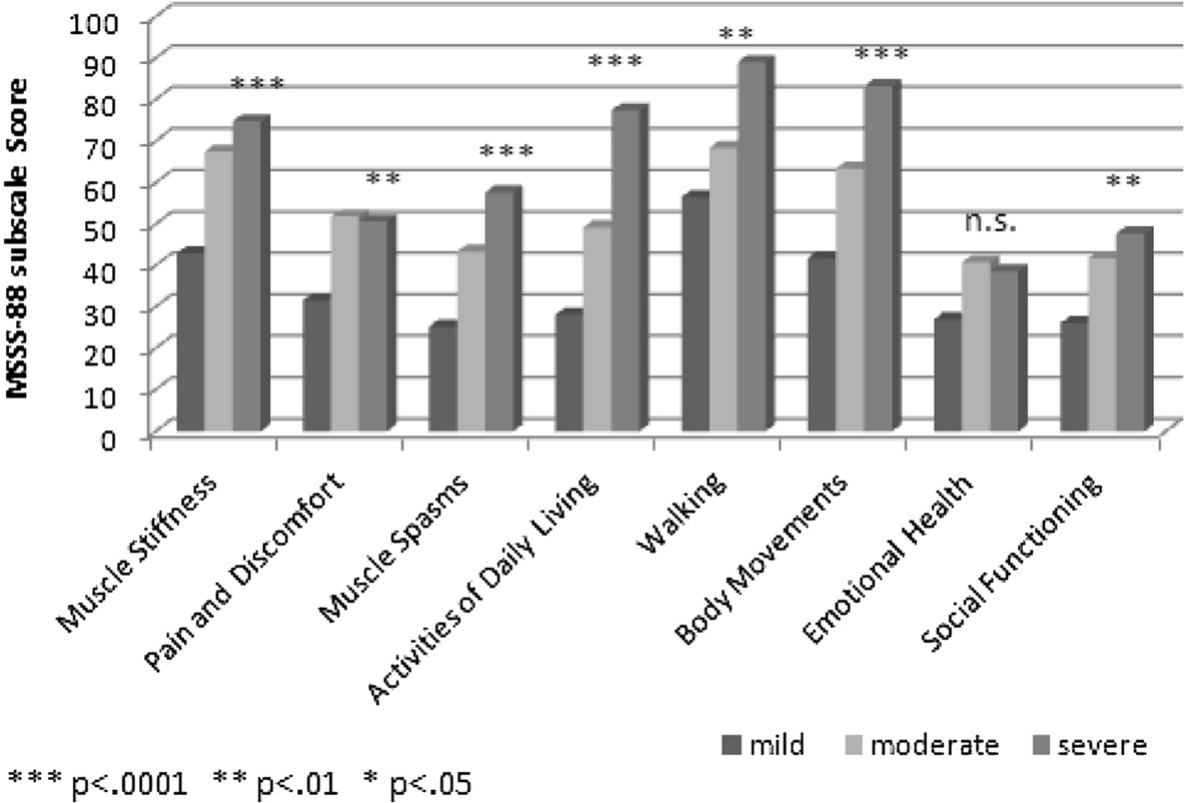
**Mean MSSS-88 subscale score for people with mild (n = 37), moderate (n = 25), or severe spasticity (n = 23) [rated by physician].**

Concerning the *severity of spasticity*, significant differences between patients with mild, moderate or severe spasticity could be recorded for all subscales of the MSSS-88 except for ‘emotional health’ (Figure 2).

Patients with *high increase of muscle tone* (grade 3-4 on the modified Ashworth scale) reported higher impairments in almost all MSSS-88 subscales compared to patients with no or low increase of muscle tonus (grade 0-2), except for ‘emotional health’ (Table 5). For *type of spasticity*, significant differences were found. Patients with both types of spasticity (persistent, paroxysmal) reported higher impairments in five of the MSSS-88 subscales, except for ‘pain/discomfort’, ‘walking’ and ‘body movement’ (Table 5).

**Table 5:** **Known groups' validity of the MSSS-88 [at inclusion (n = 87)]**

**Subscale MSSS-88**	**Type of spasticity (physician assessment)**							**Modified Ashworth scale value group (physician assessment)**				
	**Persistent**		**Paroxysmal**		**both**		**p-value**	**0 to 2**		**3 to 4**		**p-value**
	**(n = 46)**		**(n = 31)**		**(n = 10)**			**(n = 48)**		**(n = 37)**		
	**n**	**Mean ± SD**	**n**	**Mean ± SD**	**n**	**Mean ± SD**		**n**	**Mean ± SD**	**n**	**Mean ± SD**	
Muscle Stiffness	44	53.85 ± 24.96	30	57.85 ± 25.13	10	81.50 ± 16.45	0.0067	48	46.98 ± 24.25	36	74.03 ± 17.81	<0.0001
Pain and Discomfort	43	40.35 ± 23.57	29	40.63 ± 29.40	10	58.15 ± 27.83	0.1434	47	36.84 ± 26.54	35	50.39 ± 24.95	0.0215
Muscle Spasms	45	34.47 ± 20.56	30	40.70 ± 26.51	10	56.90 ± 24.40	0.0243	48	29.09 ± 21.24	37	52.57 ± 20.96	<0.0001
Activities of Daily Living	44	44.20 ± 28.82	28	42.53 ± 34.60	10	70.91 ± 27.37	0.0351	47	32.03 ± 28.32	35	66.84 ± 24.38	<0.0001
Walking	39	64.87 ± 24.16	23	60.74 ± 28.38	6	81.67 ± 20.30	0.2068	46	57.91 ± 25.99	22	79.70 ± 17.70	0.0007
Body Movements	45	55.70 ± 25.68	28	56.17 ± 32.13	9	79.46 ± 21.84	0.0608	48	46.79 ± 27.31	34	74.96 ± 20.83	<0.0001
Emotional Health	45	31.15 ± 22.90	30	32.65 ± 26.31	9	53.56 ± 28.48	0.0481	48	30.49 ± 25.31	36	38.89 ± 25.04	0.1343
Social Functioning	43	34.55 ± 25.96	30	32.60 ± 27.39	10	56.67 ± 30.63	0.0459	47	29.37 ± 26.45	36	45.83 ± 26.98	0.0066

Mean MSSS-88 subscale scores for type of spasticity and muscle tonus [rated by physician].

### Responsiveness

Responsiveness was explored in a total of 66 people: 24 treated with cannabinoid spray (“treated group”), 42 people not treated (“standard group”). As predicted, change of spasticity scores and beneficial effect sizes were greater in the treated than the standard group (Table 6). In the treated group significant changes with a moderate effect size were found for ‘muscle spasms’, ‘emotional health’ and ‘pain/discomfort’; same was found for the NRS. In the standard group, no significant changes could be detected in the NRS spasticity scale or in any MSSS-88 subscale. Therefore, sensitivity to change of the MSSS-88 scale could be demonstrated.

**Table 6:** **Responsiveness (MSSS-88, NRS)**

**MSSS-88 Subscale**	**Patients treated with cannabinoid oromucosal spray (n = 24)**						**Patients not treated with cannabinoid oromucosal spray (n = 42)**					
	**n**	**Baseline**	**Follow-Up**	**Change baseline to Follow-up**			**n**	**Baseline**	**Follow-Up**	**Change baseline to Follow-up**		
		**M ± SD**	**M ± SD**	**Mean Difference (SD)**	**p- value** ^**+**^	**Cohen d**		**M ± SD**	**M ± SD**	**Mean Difference (SD)**	**p- value** ^**+**^	**Cohen d**
Muscle Stiffness	22	76.55 ± 17.58	70.74 ± 14.25	-5.81 (15.08)	0.0852	0.39	42	48.74 ± 24.60	48.45 ± 19.88	-0.28 (16.92)	0.9143	0.02
Pain and Discomfort	21	56.97 ± 27.25	46.80 ± 22.74	-10.16 (19.97)	0.0302	0.51	42	34.87 ± 24.36	35.98 ± 20.84	1.11 (17.80)	0.6873	0.06
Muscle Spasms	23	56.48 ± 18.21	41.69 ± 14.04	-14.79 (22.45)	0.0045	0.66	41	32.20 ± 21.47	29.97 ± 17.11	-2.23 (17.49)	0.4189	0.13
Activities of Daily Living	24	67.30 ± 26.55	66.49 ± 26.15	-0.80 (12.65)	0.7581	0.06	38	34.99 ± 30.53	34.94 ± 26.98	-0.06 (17.05)	0.9840	0.00
Walking	15	72.44 ± 21.10	68.67 ± 21.63	-3.78 (20.47)	0.4864	0.18	38	60.10 ± 27.02)	59.47 ± 28.45	-0.62 (21.07)	0.8562	0.03
Body Movements	22	74.10 ± 22.20	66.36 ± 22.23	-7.74 (17.01)	0.0446	0.46	41	49.31 ± 28.93	45.97 ± 24.53	-3.33 (20.39)	0.3014	0.16
Emotional Health	22	43.50 ± 24.11	32.05 ± 23.84	-11.45 (22.30)	0.0253	0.51	42	30.43 ± 25.26	28.33 ± 24.92	-2.11 (19.49)	0.4876	0.11
Social Functioning	22	49.51 ± 28.48	41.72 ± 24.42	-7.79 (18.97)	0.0677	0.41	41	30.05 ± 25.27	29.81 ± 22.41	-0.25 (23.15)	0.9459	0.01
NRS spasticity	24	6.71 ± 1.55	5.96 ± 1.71	-0.75 (1.26)	0.0078	0.60	41	3.98 ± 2.25	3.71 ± 2.11	-0.27 (1.64)	0.3022	0.16

^+^T- test.

## Discussion

The linguistic validation and cross-cultural adaptation of the English MSSS-88 questionnaire into German underwent forward/backward translations, reconciliation, discussion with clinical MS-experts, CD with MS patients and psychometric testing of the MSSS-88 questionnaire in MS patients with different severities of spasticity as required by published guidelines for translations of PRO instruments [43]. Floor effects were found for the subscales ‘walking’ and ‘body movements’, indicating that patients reported very high impairments in these subscales. Internal consistency showed values for all subscales above Cronbach’s α = 0.90, mirroring the results from the original data, and high ICCs were found for re-test reliability testing after 1 month follow-up for all subscales above r = 0.61, except for ‘muscle spasm’. Convergent validity was proven, with acceptable correlations with the generic EQ-5D-3L questionnaire) and good correlations for the disease-specific MSQoL-54 questionnaire. Discriminant validity could be demonstrated with low correlations with the symptom-specific WEIMuS Inventory. The MSSS-88 could significantly discriminate between clinical subgroups of spasticity both for self-rated (NRS assessment) and physician-rated severity. A higher impairment could be demonstrated in those patients suffering from both types of spasticity - persistent and paroxysmal. Sensitivity to change could be proven for ‘treated’ patients using the cannabinoid oromucosal spray in four out of eight MSSS-88 subscales (‘muscle spasm’, ‘emotional health’, ‘pain/discomfort’ and ‘body movement’). This reduction was mirrored by the difference in the self-rated severity of spasticity (p = 0.0078). By contrast, no significant difference could be found in the ‘standard group’ not using treatment. It should be mentioned that patients treated with cannabinoid oromucosal spray had at inclusion already higher impairments in the subscale ‘muscle stiffness’ compared to patients in the untreated group, which can be explained with the requirements of treatment using cannabinoid oromucosal spray only in patients with moderate to severe spasticity. Furthermore in the group of untreated patients 72.5% had mild spasticity at inclusion compared to none of the patients in the treated group, while 41.7% of the treated group suffered from severe spasticity compared to only 15.7% in the untreated group.

The German MS patients were comparable to the original English study sample in terms of demographic characteristics (mean age 50 vs. 54 years, female 62% vs. 63%). By contrast, they differed related to clinical data (duration of MS from onset 13 vs. 21 years, unaided walking 33% vs. 5.4%, Mean Modified Ashworth score 2 vs. 0.95). In the German study, spasticity was assessed globally by means of the Modified Ashworth Scale, not specific for different muscles, while in the original CAMS study, spasticity was assessed across 20 muscles by means of the Modified Ashworth Scale. In attempting to compare our findings for the Modified Ashworth Scale with the original data of the CAMS study, where data were derived in different ways, we divided the mean value of the Modified Ashworth scale of (M = 19) by 20 based on the algorithm used in the CAMS study, with a possible range of 0-80 (compared to the range of the Modified Ashworth Scale of 0-4 in the German cohort). As one would expect, the findings in the CAMS study are lower in the Modified Ashworth scale than the German MOVE2 study, since here an unspecified global assessment of spasticity was done. Moreover, it should be taken into consideration for the interpretation of the results that the (Modified) Ashworth Scale has been demonstrated not to be a valid and reliable instrument for the assessment of the degree of spasticity [44,45]. PROs are considered a necessary component of health outcome measures comprising the impact of the disease or the treatment on different aspects of life [46] and therefore should not only be implemented in clinical trials, but also in routine care [47]. Depending on the purpose of a specific study or the needs of PRO assessment in routine care, the MSSS-88 scale can be used together with other instruments assessing different aspects important for patients with MS, such as self management [48], functioning [37] and fatigue [35].

A limitation of this validation study might be the relatively low number of enrolled patients (n = 87) for the psychometric testing of the newly-translated MSSS-88 questionnaire. This is partly due to the fact that from the initially planned ten study centers two did not participate in the study because of organizational reasons. Unfortunately not all participating patients filled in the re-test questionnaire (n = 66): from those 36 patients starting to use cannabinoid oromucosal spray at inclusion, only 24 patients filled in the re-test of the MSSS-88 questionnaire. In the current study, we did not evaluate the measurement equivalence, due to two reasons. First, the number of enrolled patients was relatively low, so that we could not do these analyses and second the aim of this paper was the linguistic and psychometric validation of the newly translated MSSS-88. Without this measurement, a meaningful comparison of results across countries is difficult [43]. In general, we would recommend testing measurement equivalence, which is defined as “whether or not, under different conditions of observing and studying phenomena, measurement operations yield measures of the same attribute” [49], if testing of cross-cultural differences is intended. Although the number of enrolled patients (n = 87) was lower than expected, the response rate was higher (87%) than in the original development study of the MSSS-88 scale (78%) [26], where in total 259 patients were enrolled. Since the psychometric characteristics of the original MSSS-88 scale could already be clearly and homogenously verified in this relatively limited cohort of MS patients, we do not expect differences in a bigger cohort and therefore would like to apply our findings to the German MS-population. Moreover, Hobart published recently an estimation of necessary sample size for reliability and validity testing in neurology and concluded that reliability estimates are stable in magnitude in sample sizes of a minimum of 20, and validity estimates are stable in samples of n ≥ 80 [50], which confirms our interpretation that our results can be generalized.

## Conclusion

This new translation of the MSSS-88 questionnaire into German was easily understood by MS patients and proven to be a reliable and valid instrument for the assessment of the impact of spasticity on MS patients. It showed excellent psychometric characteristics - similar to the original English instrument - although its measurement equivalence could not be tested in the frame of the current study. Moreover, it could be shown that the new instrument is sensitive to changes and therefore it can be administered in clinical trials aiming to track the evolution of MS-spasticity.

## Abbreviations

CD: Cognitive debriefing
CDF: Chart documentation form
EDSS: Expanded disability status scale
FU1: Follow-up visit after one month
HRQoL: Health-related quality of life
ICC: Intra- class correlation coefficient
MS: Multiple sclerosis
MSQoL–54: Multiple sclerosis Quality of life instrument
MSSS-88: Multiple sclerosis spasticity scale
NRS: Numeric rating scale
PRO: Patient reported outcomes
PE: Psychometric evaluation
QoL: Quality of life
SD: Standard deviation
WEIMuS: Würzburger fatigue inventory for MS
